# *CXCR2P1* enhances the response of gastric cancer to PD-1 inhibitors through increasing the immune infiltration of tumors

**DOI:** 10.3389/fimmu.2025.1545605

**Published:** 2025-03-19

**Authors:** Xinchun Wu, Sen Hou, Yingjiang Ye, Zhidong Gao

**Affiliations:** ^1^ Department of Gastrointestinal Surgery, Peking University People`s Hospital, Beijing, China; ^2^ Laboratory of Surgical Oncology, Peking University People`s Hospital, Beijing, China; ^3^ Beijing Key Laboratory of Colorectal Cancer Diagnosis and Treatment Research, Peking University People’s Hospital, Beijing, China

**Keywords:** *CXCR2P1*, prognosis, immune microenvironment, PD-1 inhibitor, microRNAs

## Abstract

**Background:**

Recent years, immunotherapy has emerged as a pivotal approach in cancer treatment. However, the response of gastric cancer to immunotherapy exhibits significant heterogeneity. Therefore, the early identification of gastric cancer patients who are likely to benefit from immunotherapy and the discovery of novel therapeutic targets are of critical importance.

**Materials and methods:**

We collected data from European Nucleotide Archive (ENA) and Gene Expression Omnibus (GEO) databases. In project PRJEB25780, we performed WGCNA analysis and Lasso regression and chose *CXCR2P1* for the subsequent analysis. Then, we compared the expression difference of *CXCR2P1* among different groups. Kaplan-Meier curve was used to analyze the prognostic value of *CXCR2P1*, which was validated by project IMvigor210 and GEO datasets. ESTIMATE and CIBERSORT algorithm were used to evaluate the reshaping effect of *CXCR2P1* to immune microenvironment of tumor. Differentially expressed genes (DEG) analysis, enrichGO analysis, Gene Set Enrichment Analysis (GSEA) and co-expression analysis were used to explore the cell biological function and signaling pathway involved in *CXCR2P1.*

**Results:**

WGCNA identified *CXCR2P1* as a hub gene significantly associated with immune response to PD-1 inhibitors in gastric cancer. *CXCR2P1* expression was elevated in responders and correlated with better prognosis. Functional analysis revealed its role in reshaping the tumor immune microenvironment by promoting immune cell infiltration, including M1 macrophages, activated *CD4*+ T cells, and follicular helper T cells. *CXCR2P1* enhanced antigen presentation via the MHC-II complex, influenced key immune pathways, such as Toll-like receptor signaling and T-cell activation, which led to the up-regulation of expression of PD-L1. GSEA showed *CXCR2P1* were correlated with microRNAs. Through DEG analysis and expression analysis, *MIR215* was identified as a potential direct target of *CXCR2P1*.

**Conclusion:**

High expression of CXCR2P1 is correlated with better response to PD-1 inhibitor. It reshapes the immune microenvironment by increasing immune infiltration and changing the fraction of immune cells. In tumor immune microenvironment, *CXCR2P1* can promote inflammation, enhance antigen presentation and activate the PD-1/PD-L1-related signaling pathway, which might be achieved by *CXCR2P1*-*MIR215* axis.

## 1 Introduction

Gastric cancer is the fifth most common cancer in the world and causes about 770 000 deaths every year. Of note, East Asia region has the highest gastric cancer incidence (22.4/100,000 inhabitants) and mortality (14.6/100,000) (1). In recent years, immunotherapy has become a significant adjuvant treatment for gastric cancer, with programmed cell death-1 (PD-1) inhibitors emerging as an effective option, particularly for advanced or metastatic cases (2). Studies have demonstrated that PD-1 inhibitors, including nivolumab and pembrolizumab, improve patient prognosis, leading to their incorporation into the National Comprehensive Cancer Network (NCCN) guidelines (3–5). However, not all gastric cancer patients can benefit from PD-1 inhibitors.The response rate of tumors to PD-1 inhibitors varies with the microsatellite stability, the degree of immune cell infiltration, the level of PD-L1 expression and so on (6). It is reported that unselected patients who receive anti-PD-1 and anti-PD-L1 immunotherapy have a total response rate of only approximately 20% (7). Therefore, it is important to identify patients who may benefit from PD-1 inhibitors before or early in the treatment of gastric cancer.

Finding out which genes are involved in the PD-1 signaling pathway, analyzing the heterogeneity of the expression of these genes are the key to explore the interaction mechanism between tumor and immune cells, so that we can develop more targeted therapies. By analyzing genes that were differentially expressed between gastric cancer patients who responded well to PD-1 inhibitors and those who did not respond to PD-1 inhibitors, we find *C-X-C Motif Chemokine Receptor 2 Pseudogene 1 (CXCR2P1)*, a pseudogene encoding long non-coding RNA (lncRNA) which may affect cell phenotype through microRNA according to the previous research, plays a key role in the response of gastric cancer cells to PD-1 inhibitor (8). However, based on our investigation so far, only a few researchers have mentioned *CXCR2P1* in their studies. The role of *CXCR2P1* in tumor development and its effect on tumor immune microenvironment still remains unknown (8–12).

In this study, we compared expression levels of *CXCR2P1* in different groups from different public databases, validated the potential of *CXCR2P1* as a predictor of prognosis in gastric cancer patients receiving PD-1 inhibitors. Meanwhile, we analyzed the correlation between *CXCR2P1* and tumor microenvironment. Finally, the signaling pathways involved in *CXCR2P1* in gastric cancer cells were analyzed by differentially expressed genes analysis, enrichment analysis and co-expression analysis. This study explored the role of *CXCR2P1* in the tumor immune response in detail, which provided a new target and idea for the immunotherapy of gastric cancer.

## 2 Materials and methods

### 2.1 Data and resources

Aspera tools were used to download fastq sequencing data of project PRJEB25780 (gastric cancer immunotherapy data set) from the European Nucleotide Archive (ENA) database (https://www.ebi.ac.uk/ena). Subreads, HISAT2 (version 2.2.1), and samtools were used to generate the counts matrix and Transcripts Per Kilobase of Exon Model Per Million Mapped Reads (tpms) from fastq sequencing data for subsequent analysis. Quantile normalized (log10) Fragments Per Kilobase of Transcript Per Million Mapped Reads (fpkm) of project PRJEB25780 were downloaded from Tumor Immune Dysfunction and Exclusion (TIDE) database. Clinical information of project PRJEB25780 were downloaded from materials of Kim’s research (13). The four gastric cancer microarray datasets (GSE193453, GSE174237, GSE84433, GSE27342) and the lung cancer immunotherapy data set (GSE135222) can be downloaded from the GEO database (https://www.ncbi.nlm.nih.gov/geo/). The expression matrix and clinical information of metastatic urothelial carcinoma immunotherapy data set (IMvigor210) were obtained through “IMvigor210CoreBiologies” R package. Gene sets of PD-1 checkpoint signaling pathway were downloaded from Kyoto Encyclopedia of Genes and Genomes (KEGG) database (https://www.kegg.jp/kegg/). All tpms matrix used for analysis were normalized by log_2_.

### 2.2 Gene screening

On project PRJEB25780, Weighted Correlation Network Analysis (WGCNA) was performed by “WGCNA” R package for seeking gene modules with highly synergistic changes in expression. The response to PD-1 inhibitor of gastric cancer patients were regarded as the clinical phenotypes for subsequent WGCNA. We first used the hclust function to cluster a total of 45 samples, excluded the outlier sample, and used the remaining samples for network topology analysis to determine the appropriate soft threshold. Then, gene network modules were constructed by the “blockwiseModules” function with the minimum number of 30 genes in each module. Each module was assigned a color, and genes that could not be clustered into other modules were uniformly assigned gray. The eigengenes of each module were calculated by the “moduleEigengenes” function. Finally, based on the eigengenes of each module, the correlation between the modules and the response to PD-1 was calculated. A heat map illustrating the module-phenotype correlations was generated, and the module showing the strongest correlation was chosen for further analysis.

Gene significance (GS) is defined as the absolute value of the correlation between a gene and the phenotypic trait. Module membership (MM) is defined as the correlation between the eigengene of a module and the gene expression within the module. In this study, we used the condition of GS > 0.2 and MM > 0.8 as the screening criteria for hub gene of the module we chose above.

Normalized expression matrix of hub genes screened by WGCNA was performed Least Absolute Shrinkage and Selection Operator regression (Lasso) by the “glmnet” R package. Next, cross-validation was used to select the optimal regularization parameter λ to maximize the prediction performance of the model. After obtaining the best model, the features with nonzero coefficients were retained for subsequent analysis.

### 2.3 Clinical features and *CXCR2P1*

Expression difference of *CXCR2P1* between responders and non-responders subgroups were described in project PRJEB25780 and IMvigor210. Responder was defined as complete response (CR) or partial response (PR) and non-responder was defined as stable disease (SD) or progressive disease (PD) on clinical evaluation after treatment with PD-1 inhibitors. *CXCR2P1* expression between gastric cancer tissue and normal tissue were also analyzed in four microarray datasets (GSE193453, GSE174237, GSE84433, GSE27342).

### 2.4 Kaplan-Meier curve

The prognostic value of *CXCR2P1* was validated by overall survival (OS) from project IMvigor210 and progression free survival (PFS) from project GSE135222. OS was defined as the time from surgery to death due to any cause, PFS was defined as the time from surgery to recurrence, metastasis, or death due to disease.

### 2.5 Immune microenvironment analysis

Immune score was calculated based on the proportion of immune cells by ESTIMATE algorithm. The Immune score of *CXCR2P1*-High group and *CXCR2P1*-Low group were compared in patients of project PRJEB25780. Meanwhile, the fraction of immune cells of each tumor sample in project PRJEB25780 was estimated by CIBERSORT algorithm. All analysis in this section were performed using normalized tpms matrix.

### 2.6 Differentially expressed genes analysis

Counts matrix of PRJEB25780 was used for differentially expressed genes (DEGs) analysis by the “DESeq2” R package with the filter criteria of |log_2_Foldchange (FC) | > 1 and false discovery rate (FDR) < 0.05. In the DEGs analysis for *CXCR2P1*, patients were divided into high-expression and low-expression groups based on the median expression levels. In the DEGs analysis for Response, patients were divided into Responders and Non-Responders based on their response to PD-1 inhibitor.

### 2.7 Gene enrichment analysis

To explore the potential signaling pathway involved in *CXCR2P1*, we conducted Gene Set Enrichment Analysis (GSEA) and EnrichGO Analysis for DEGs of *CXCR2P1*. EnrichGO analysis was performed by R package “clusterProfiler”. Reference gene sets of GSEA included hallmark, c2wikipathways, c2KEGG and c5go. The screening conditions were |normalized enrichment score (NES)| > 1 and p value < 0.05.

### 2.8 Co-expression analysis

DEGs of *CXCR2P1* were conducted correlation analysis with the expression of *CXCR2P1*. DEGs with pearson correlation coefficient > 0.3 and p value < 0.05 were selected to construct the co-expression network with *CXCR2P1* by the “igraph” R package. The network was then modified in Cytoscape software (version 3.10.2).

### 2.9 Statistical analysis

In this study, all statistical analyses were performed using R software (version 4.3.2). The comparison of the expression levels of DEG-microRNAS between different groups was performed by Mann-Whitney test, except that all comparisons between the two groups were performed by two-tailed Student t test. Survival analysis was conducted by Kaplan-Meier curve. Pearson’s analysis was used for correlation analysis between modules and response to PD-1 inhibitor or between expression of *CXCR2P1* and DEGs. Spearman’s analysis was used for correlation analysis which generated scatter plots. P value of 0.05 was considered statistically significant (* means p < 0.05, ** means p < 0.01, *** means p < 0.001).

## 3 Results

### 3.1 *CXCR2P1* is one of hub genes for immune response of gastric cancer

**Figure 1:**
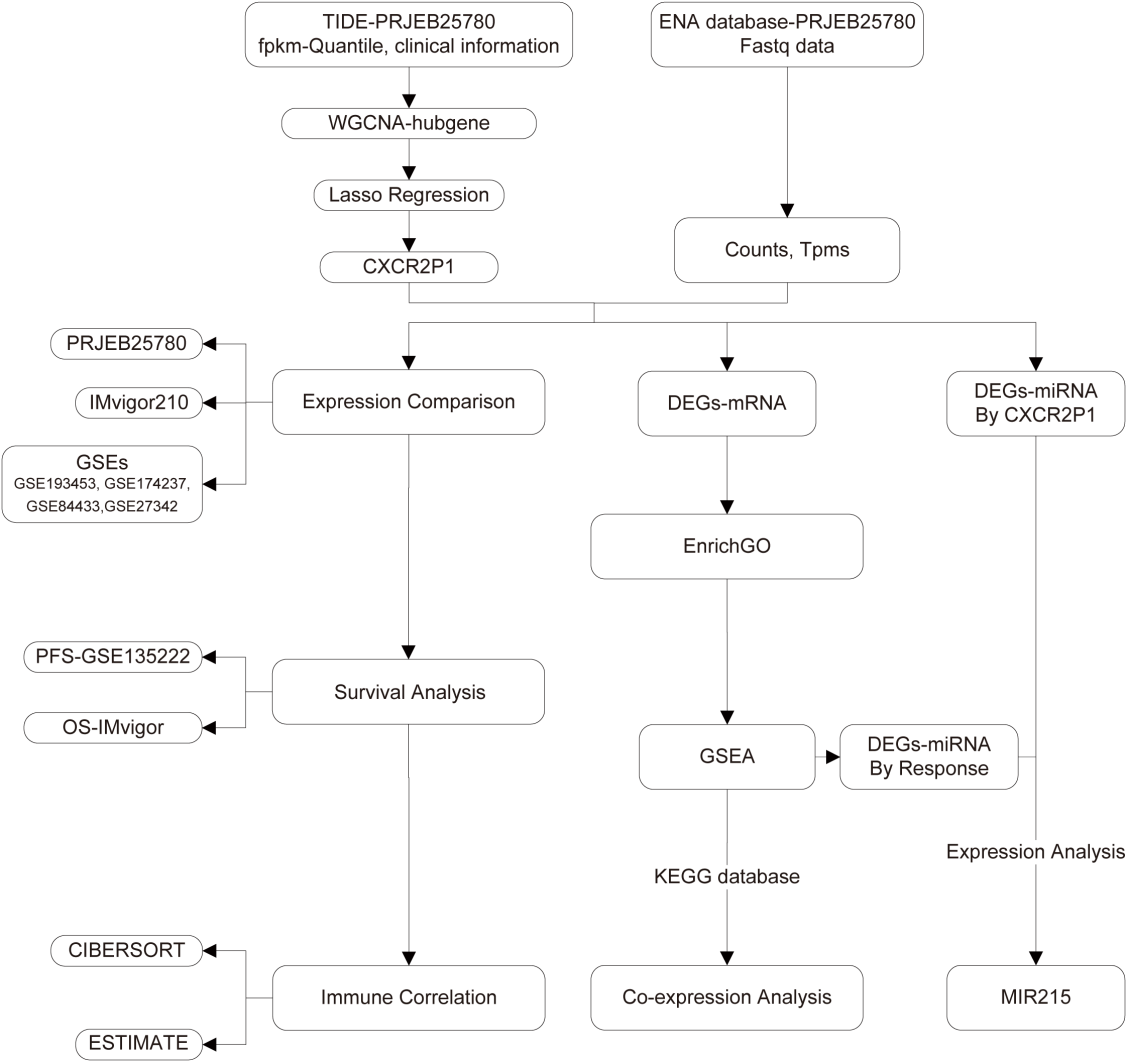
Research flow chart.

**Figure 2:**
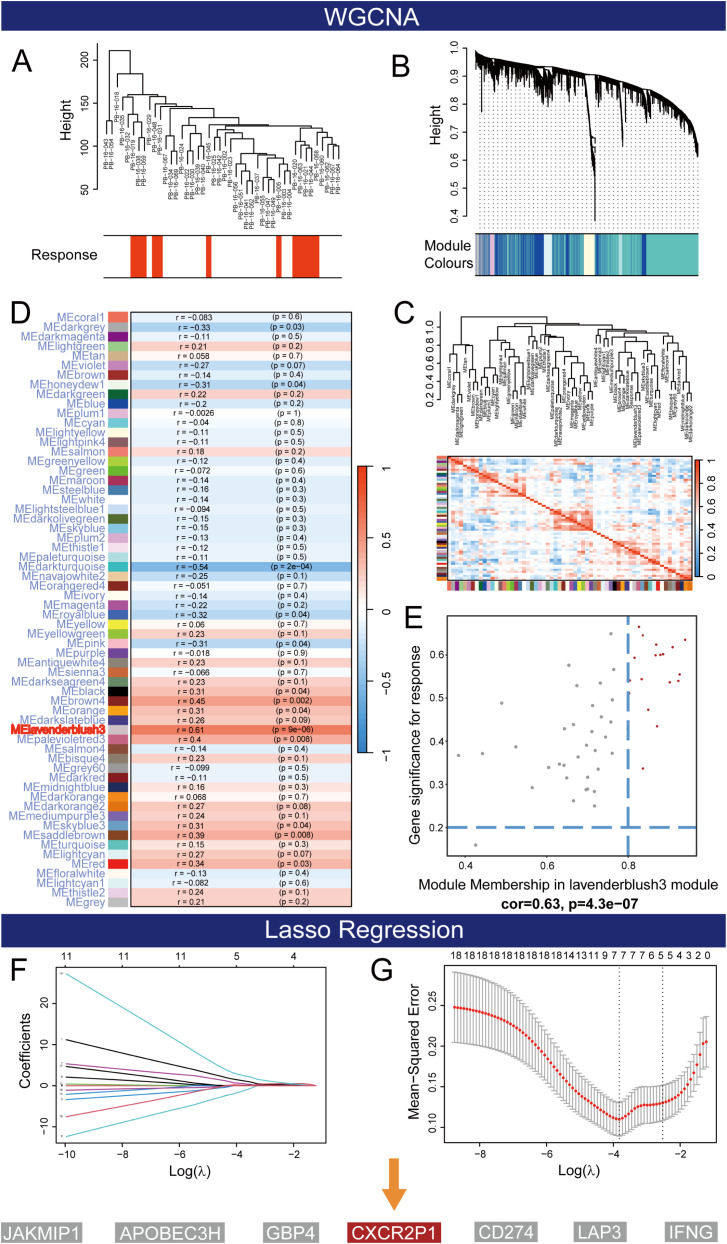
The process of screening for hub genes. **(A)** Clustering dendrogram of 44 samples with exclusion of PB-16-066. **(B)** Dendrogram of all differentially expressed genes clustered based on the measurement of dissimilarity. **(C)** Correlations and Clustering among modules. **(D)** Correlation between response to PD-1 inhibitor and modules. **(E)** Screening for hub genes within lavenderblush3 module. **(F)** Lasso coefficient path plots for the 18 hub genes. **(G)** Cross validation curves for lasso regression.

**Figure 1** shows the flow chart of this study. With row data processing, we obtained the transcriptome expression matrix and clinical information of project PRJEB25780. In WGCNA analysis, we first clustered samples based on their normalized expression matrix and response to PD-1 inhibitor and exclude the sample that was apparently outlier (**Figure 2A**, sample code: PB-16-066). Then, a network consisting of highly cooperative gene modules was constructed by network topology analysis (**Figure 2B**). In the end, we got 61 effective modules in total with different colors. **Figure 2C** shows the correlation among the modules. Next, we conducted correlation analysis between modules and the clinical phenotype (Responder and Non-Responder) and found module lavenderblush3 had the highest correlation with the immune response of the tumor (r_pearson_ = 0.61, p = 0.000009, shown as **Figure 2D**).

After loading the lavenderblush3 module, we obtained a total of 53 intra-module genes. The scatter plot shown in **Figure 2E** was used to represent the relationship between GS and MM of each gene in the lavenderblush3 module, whose fitting curve showed a significant correlation between GS and MM (r_pearson_ = 0.63, p = 0.00000043). Genes with GS > 0.2 and MM > 0.8 were selected as hub genes. Finally, we obtained 18 hub genes of lavenderblush3 module (*GBP4*, *GBP5*, *JAKMIP1*, *APOBEC3H*, *GBP1*, *GBP2*, *CD274*, *IFNG*, *CXCR2P1*, *IDO1*, *CXCL10*, *GBP1P1*, *CXCL9*, *LAP3*, *CXCL11*, *TAP1*, *WARS*, *UBE2L6*).

To reduce the collinearity among hub genes, the lasso regression analysis was performed. As the penalty function (log λ) increases, the regression coefficient of each variable in the model gradually converge to 0 (**Figure 2F**). In the cross-validation, when the number of variables in the model was reduced to 7 (*GBP4*, *JAKMIP1*, *IFNG*, *CXCR2P1*, *LAP3*, *CD274*, *APOBEC3H*), the mean square error of the model was reduced to the lowest point, and the fitting effect of the equation reached the best (**Figure 2G**). Since the mechanism of how *CXCR2P1* affects cell phenotype is still unclear, it is selected for subsequent analysis.

### 3.2 *CXCR2P1* is highly expressed in responders and associated with good prognosis

**Figure 3:**
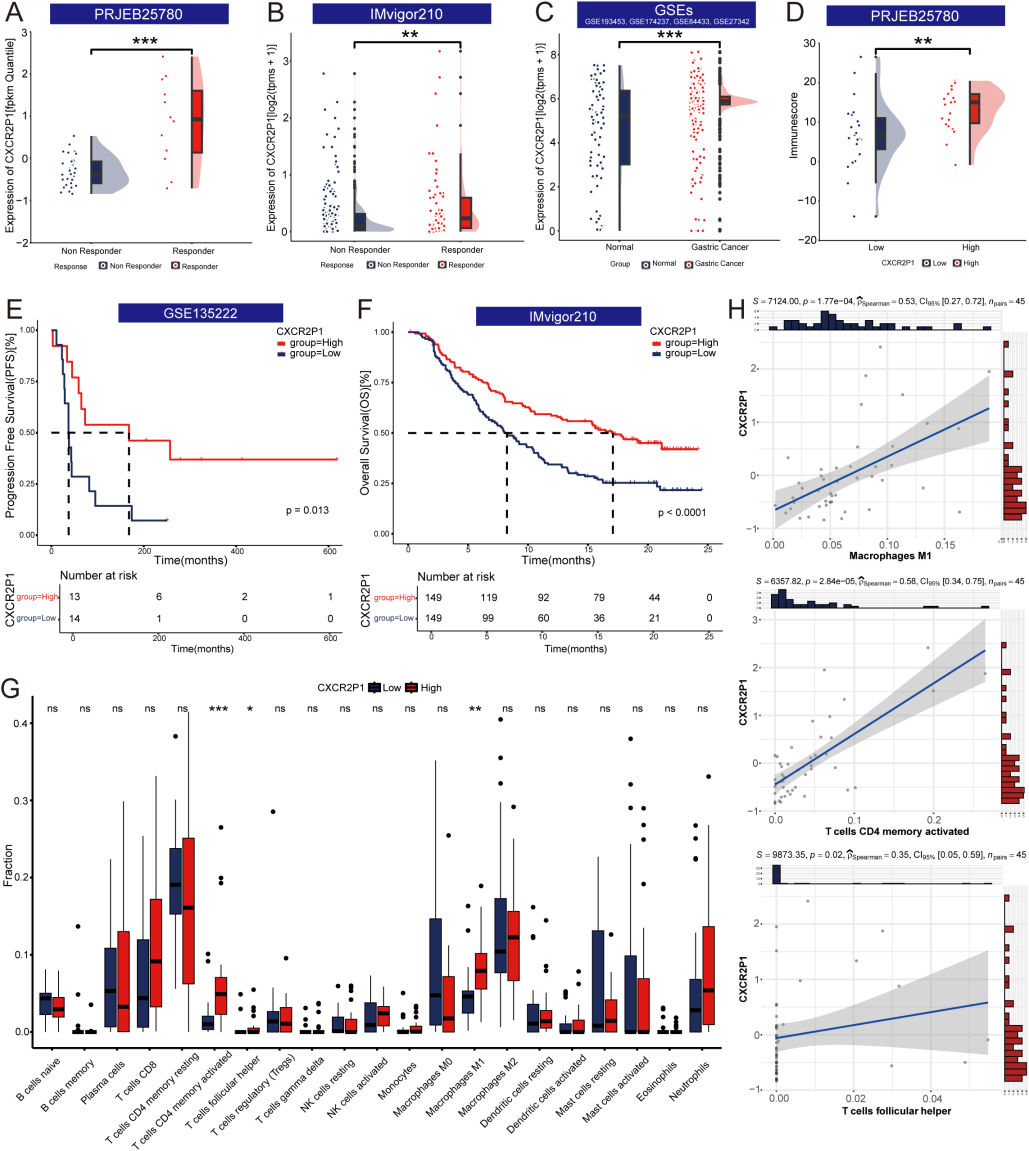
Exploration of the function of *CXCR2P1*. **(A)** Comparison of expression of *CXCR2P1* between responders and non-responders in project PRJEB25780. **(B)** Comparison of expression of *CXCR2P1* between responders and non-responders in project IMvigor210. **(C)** Comparison of expression of *CXCR2P1* between gastric cancer tissue and normal gastric mucous tissue in GEO datasets. Four GEO datasets are GSE193453, GSE174237, GSE84433 and GSE27342. **(D)** Comparison of immunescore between *CXCR2P1*-High group and *CXCR2P1*-Low group. **(E)** - **(F)** Kaplan-Meier Curve of survival analysis of *CXCR2P1*. High Expression of *CXCR2P1* had prolonged PFS in GSE135222 and prolonged OS in project IMvigor210. **(G)** Comparison of the fraction of immune cells between *CXCR2P1*-High group and *CXCR2P1*-Low group. **(H)** Correlation between the *CXCR2P1* and Macrophages (M1, ρ_Spearman_ = 0.53, p = 0.000177), activated CD4+ memory T cells (ρ_Spearman_ = 0.58, p = 0.0000284), and follicular helper T cells (ρ_Spearman_ = 0.35, p = 0.02). PFS, progression free survival; OS, overall survival; *p < 0.05; **p < 0.01; ***p < 0.001.

The prognosis of patients with gastric cancer is highly correlated with their response to the PD-1 inhibitor. Trough analyzing different data sets, expression of *CXCR2P1* was found higher in responders than which in non-responders in both project PRJEB25780 (**Figure 3A**, p < 0.001) and project IMvigor210 (**Figure 3B**, p < 0.01). In four GEO datasets, expression of *CXCR2P1* was higher in gastric cancer tissue than which in normal tissue (**Figure 3C**, p < 0.001). Through Kaplan-Meier curves, we found *CXCR2P1*-high group had prolonged OS in patients with metastatic urothelial carcinoma (**Figure 3F**, p < 0.0001) and prolonged PFS in patients with lung cancer (**Figure 3E**, p = 0.013) than those in *CXCR2P1*-low group. Based on results above, patients with higher expression of *CXCR2P1* might possess better prognosis.

### 3.3 *CXCR2P1* reshape the tumor immune microenvironment

In project PRJEB25780, ESTIMATE analysis showed *CXCR2P1*-high group had higher immune score, which revealed that high expression of *CXCR2P1* increased the degree of infiltration of immune cells to tumors (**Figure 3D**). CIBERSORT algorithm was used to evaluate the abundance of immune cells and compared the fractions of different immune cells between *CXCR2P1*-high and *CXCR2P1*-low group (**Figure 3G**). Result show that the fraction of Macrophages (M1), activated *CD4*+ memory T cells, and follicular helper T cells was higher in *CXCR2P1*-high group than which in *CXCR2P1*-low group. Meanwhile, the linear equation of scatter plots shown as **Figure 3H** revealed a significant positive correlation between *CXCR2P1* expression and the fraction of Macrophages (M1, ρ_Spearman_ = 0.53, p = 0.000177), activated *CD4*+ memory T cells (ρ_Spearman_ = 0.58, p = 0.0000284), and follicular helper T cells (ρ_Spearman_ = 0.35, p = 0.02). The expression of *CXCR2P1* can influence the tumor immune microenvironment significantly.

### 3.4 *CXCR2P1* is associated with antigen processing and presentation in tumor immune microenvironment

**Figure 4:**
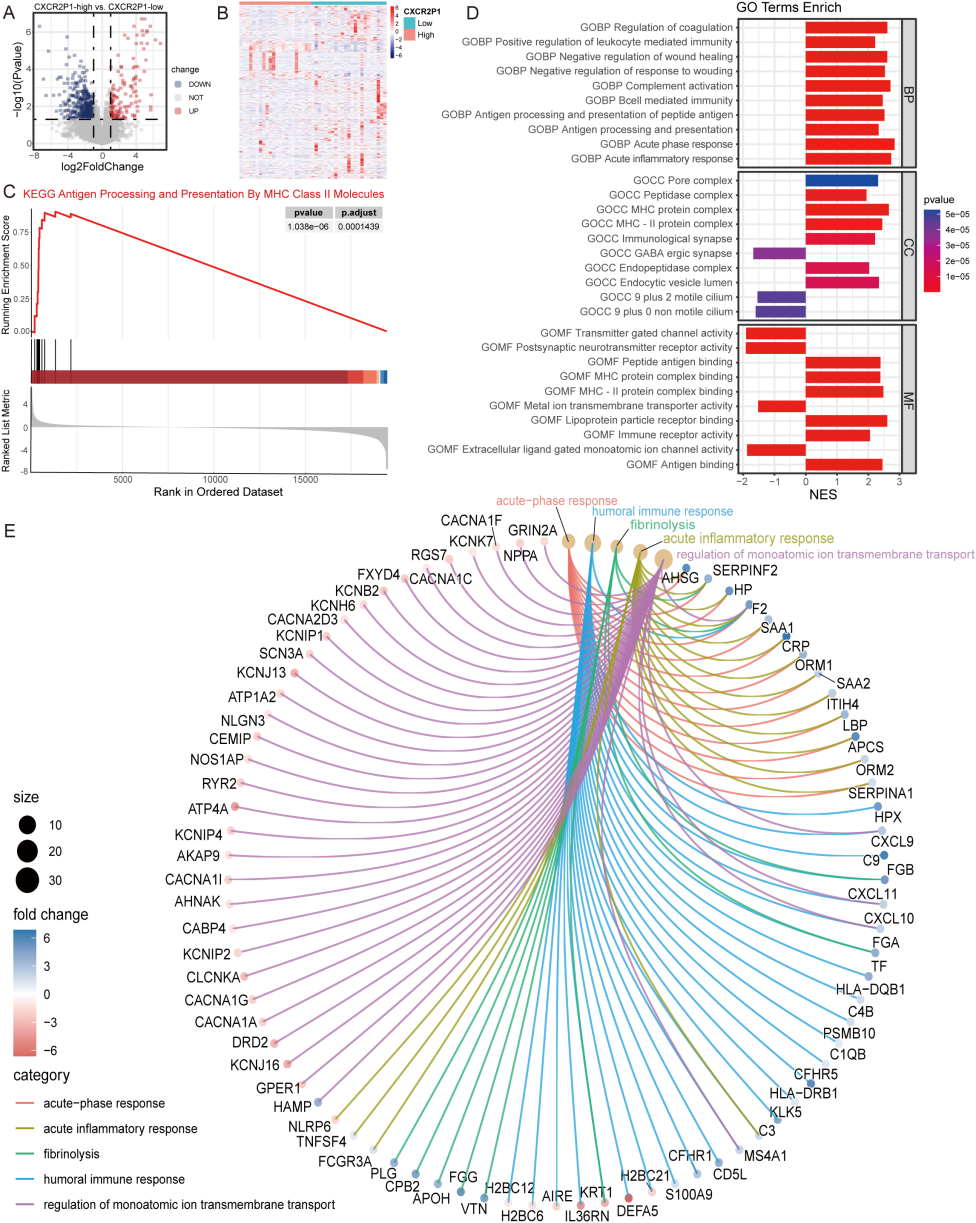
Analysis of cell biological function of *CXCR2P1*. **(A)** Volcano plot of DEG analysis by expression of *CXCRP21*. **(B)** Heatmap of DEG analysis by expression of *CXCRP21*. **(C)** GSEA analysis for DEGs of *CXCR2P1* by c2KEGG database. **(D)** GSEA analysis for DEGs of *CXCR2P1* by c5go database. **(E)** Chord diagram of top 5 enriched biological function by EnrichGO analysis for DEGs of *CXCR2P1*. BP, biological processes; CC, cellular components; MF, molecular functions; NES, normalized enrichment score.

Through differential expression analysis, 610 DEGs were found between the high- and low-expression groups of *CXCR2P1* (139 up-regulated, 471 down-regulated, as shown in **Figures 4A, B**). To explore the cellular biological function of *CXCR2P1*, EnrichGO and Gene Set Enrichment Analysis (GSEA) was performed by these DEGs. Top 5 enriched biological function by EnrichGO analysis are shown as **Figure 4E**, and 3 of them are related with biological immune response (acute-phase response, acute inflammatory response, humoral immune response). GSEA by c2KEGG database showed that *CXCR2P1* were enriched on Antigen Processing and Presentation By MHC Class II Molecules pathway (p = 0.00000103, shown as **Figure 4C**). In c5go database, biological processes (BP), cellular components (CC) and molecular functions (MF) of *CXCR2P1* were analyzed and the top 10 enriched BP, CC, and MF are listed in **Figure 4D**. Results show that the BP, CC and MF of *CXCR2P1* are all enriched in peptide antigen binding and the MHC-II protein complex, which indicate that *CXCR2P1* might increase the infiltration of immune cells in tumor issue by enhancing the antigen presentation in tumor immune microenvironment by MHC II protein complex.

### 3.5 Correlation between *CXCR2P1* and PD-1/PD-L1 signaling pathway

**Figure 5:**
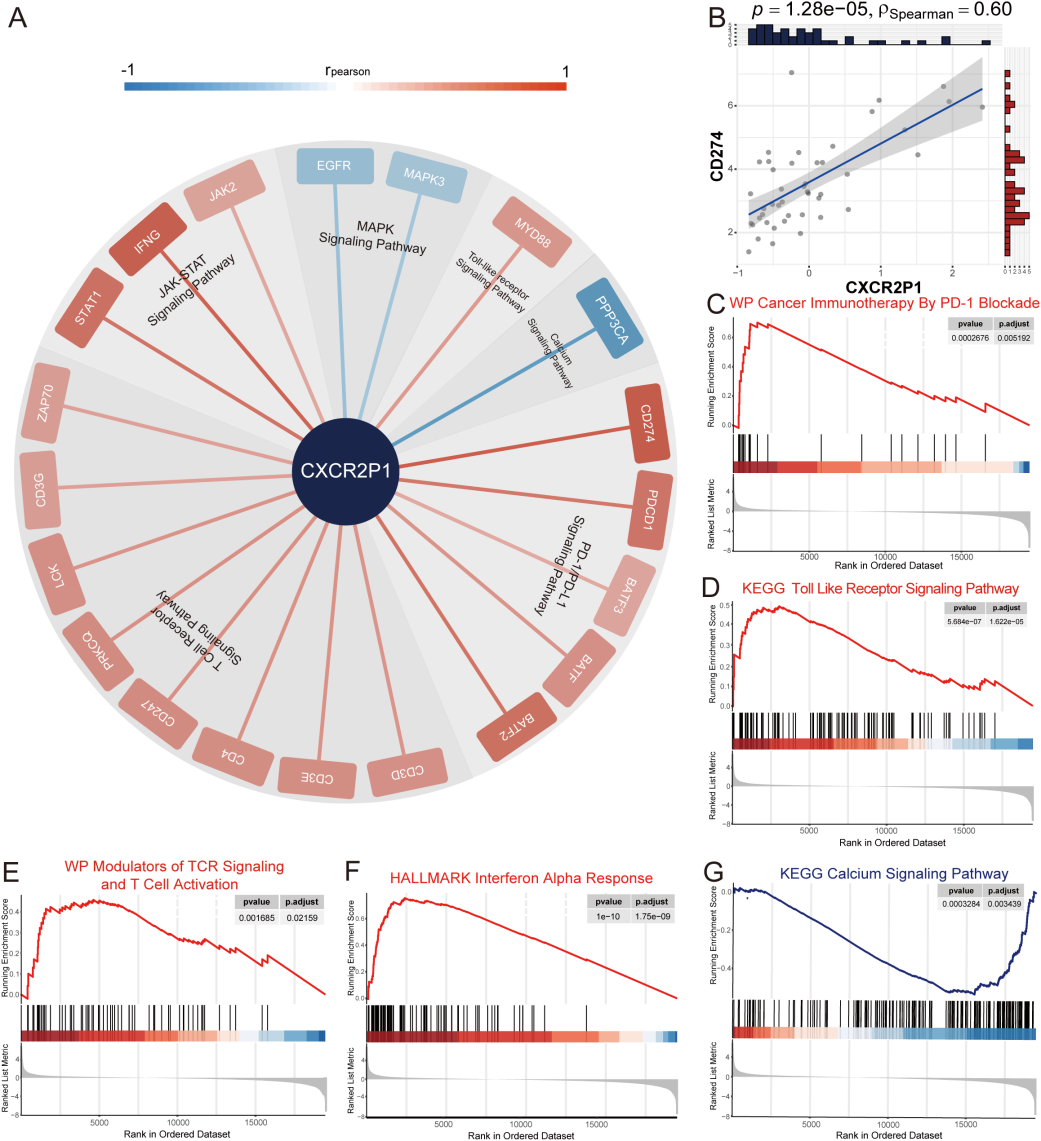
Analysis of signaling pathway of PD-L1. **(A)** Co-expression analysis between *CXCR2P1* and genes involved in PD-1/PD-L1 signaling pathway. 20 genes which were significantly associated with *CXCR2P1* (r_pearson_ > 0.3, p < 0.05) were listed around the edge of pie chart and were classified according to the signaling pathway to which they belong. **(B)** Correlation between the expression of *CXCR2P1* and *CD274*. **(C-G)** GSEA analysis for DEGs of *CXCR2P1* by c2KEGG database, Hallmark database and c2wikipathway database. Results showed that *CXCR2P1* can activate PD-1/PD-L1-related signaling pathways and suppress the activation of T cells.

To investigate the relationship between *CXCR2P1* and tumor immune evasion by PD-1/PD-L1 signaling pathway, we first analyzed the correlation between the expression of *CXCR2P1* and *CD274*. Results showed that *CXCR2P1* was highly correlated with the expression of *CD274* (ρ_Spearman_ = 0.60, p = 1.28 x 10^-5^, shown as **Figure 5B**) in tumor tissue. GSEA by c2wikipathway showed that *CXCR2P1* was significantly enriched in Cancer Immunotherapy By PD-1 Blockade (p = 2.676 x 10^-4^, shown as **Figure 5C**). Subsequently, we obtained all 73 genes involved in PD-1/PD-L1 signaling pathway from KEGG database, and analyzed the correlation between the expression of these genes and *CXCR2P1*. **Figure 5A** shows all 20 genes which are significantly related with the expression of *CXCR2P1* (p < 0.05, r_pearson_ >0.3).

Through GSEA analysis, we found 4 PD-1/PD-L1-related signaling pathways were enriched. Results showed that in tumor tissue, with the increasing expression of *CXCR2P1*, Toll Like Receptor Signaling Pathway(p = 5.684 x 10^-7^, shown as **Figure 5D**), Modulators of TCR Signaling And T Cell Activation(p = 1.685 x 10^-3^, shown as **Figure 5E**) and Interferon Alpha Signaling Pathway (p = 1.0 x 10^-10^, shown as **Figure 5F**) were significantly activated. Instead, Calcium Signaling Pathway (p = 3.284 x 10^-4^, shown as **Figure 5G**) was significantly suppressed. Our results indicated that *CXCR2P1* may indirectly up-regulate the expression of PD-L1 in tumor cells by increasing immune infiltration and enhancing the activation of T cells in the tumor tissue.

### 3.6 *CXCR2P1* influence the response of tumor cells to PD-1 inhibitor through microRNAs

**Figure 6:**
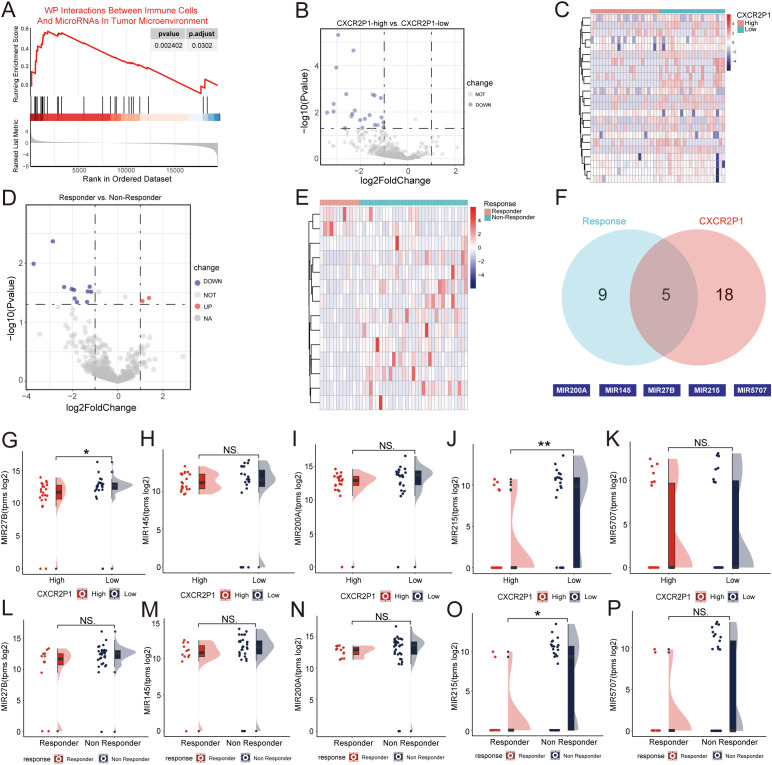
Correlation between *CXCR2P1* and microRNA. **(A)** GSEA analysis for DEGs of *CXCR2P1* by c2wikipathway database. **(B)** Volcano plot of DEG analysis for microRNAs from project PRJEB25780 by expression of *CXCRP21*. **(C)** Heatmap of DEG analysis for microRNAs from project PRJEB25780 by expression of *CXCRP21*. **(D)** Volcano plot of DEG analysis for microRNAs from project PRJEB25780 by response to PD-1 inhibitor. **(E)** Heatmap of DEG analysis for microRNAs from project PRJEB25780 by response to PD-1 inhibitor. **(F)** Venn diagram of the DEGs-microRNA by the expression of *CXCR2P1* and the response to PD-1 inhibitor. The intersection contains 5 DEGs-microRNA, including *MIR200A*, *MIR145*, *MIR215*, *MIR27B* and *MIR5707*. **(G-K)** Raincloud plots showing the comparison of the expression of the 5 DEGs-microRNA between *CXCR2P1*-high group and *CXCR2P1*-low group. **(L-P)** Raincloud plots showing the comparison of the expression of the 5 DEGs-microRNA between Responder group and Non-Responder group. NA, not available; NS, not significant; *p < 0.05; **p < 0.01; ***p < 0.001.

GSEA by c2wikipathway database showed that *CXCR2P1* was significantly enriched to Interactions Between Immune Cells And MicroRNAs In Tumor Microenvironment (2.402 x 10^-3^, shown as **Figure 6A**), which indicated that *CXCR2P1* might regulate the expression of PD-L1 through microRNAs. To further clarify which microRNAs were involved in the upstream and downstream signaling pathways of *CXCR2P1*, we first performed DEG analysis for all microRNAs in project PRJEB25780 by the expression of *CXCR2P1*, and the results showed that the expression of 23 genes was down-regulated in *CXCR2P1*-High group (**Figures 6B, C**). Then another DEG analysis was performed for all microRNAs in PRJEB25780 by the response to PD-1 inhibitor, and the results showed that 2 genes were up-regulated and 12 genes were down-regulated in Responder group (**Figures 6D, E**). By taking the intersection of the two microRNA gene sets, we obtained a total of 5 genes, including *MIR200A*, *MIR145*, *MIR215*, *MIR27B* and *MIR5707* (**Figure 6F**). The results showed that *MIR215* expression was significantly down-regulated in both *CXCR2P1*-High group (p = 0.001604, **Figure 6J**) and Responder group (p = 0.01474, **Figure 6O**). *MIR27B* was significantly down-regulated in *CXCR2P1*-High group (p = 0.0253, **Figure 6G**), but there was no significant difference between Responder and Non-Responder groups (p = 0.09778, **Figure 6L**). There was no significant difference in the expression of *MIR200A* (**Figures 6I, N**), *MIR145* (**Figures 6H, M**) and *MIR5707* (**Figures 6K, P**) between both *CXCR2P1* groups and Response groups. Our finding suggests that *MIR215* might be involved in the signaling pathway of *CXCR2P1* and serve as the mediator of *CXCR2P1* affecting cell phenotype.

## 4 Discussion

In this study, we screened out a set of genes that significantly affect the response of gastric cancer to PD-1 inhibitor by WGCNA and lasso regression for the first time. Through comprehensive analysis, we explored the potential of *CXCR2P1* as a prognostic predictor and its role in tumor immune response. Furthermore, we validated our findings using databases on PD-1 inhibitor response in other cancer types. This research analyzed the cell biological role of *CXCR2P1* firstly, explored the effect of *CXCR2P1* on the tumor immune microenvironment, and provided new insights and references for the development of targeted therapies.

The expression of *CXCR2P1* might affect the stage and prognosis of tumors. *CXCR2P1* was first mentioned in 2018 in the study of Lou et al., who found that *CXCR2P1* encodes lncRNA through RNA transcriptomics analysis and its expression was significantly up-regulated in high-grade serous ovarian cancer tissues compared with normal ovarian tissues (12). Similar results were presented in research of Ji et al. which found *CXCR2P1* was significantly up-regulated in cutaneous metastatic melanoma and high expression of *CXCR2P1* was associated with prolonged overall survival (10). Sarathi et al. confirmed that *CXCR2P1* is one of the key genes in determining the stage of hepatocellular carcinoma through differential analysis, which indicated low expression of *CXCR2P1* will lead hepatocellular carcinoma to be more invasive and metastatic (11). Our study showed that the expression of *CXCR2P1* was higher in responders and tumor tissue than those in non-responders and normal tissue. Meanwhile, in the only two databases containing both results of response to PD-1 inhibitor and survival information (lung cancer and metastatic urothelial carcinoma), *CXCR2P1* showed excellent prognostic value. Patients with high expression of *CXCR2P1* had prolonged OS and PFS. These findings validated that the high expression of *CXCR2P1* was significantly correlated with good prognosis of tumors and the prognostic value of *CXCR2P1* may be related to its effect on tumor immune response.

*CXCR2P1* may help reshape the tumor immune microenvironment. As early as 2019, Choy found *CXCR2P1* may have the ability to affect the response of tumor to immunotherapy through a pan-cancer machine-learning analysis (9). Our study validated that the tumors with higher expression of *CXCR2P1* had higher degree of immune infiltration and immune cell abundance, and the high expression of *CXCR2P1* were significantly correlated with good prognosis. The immune microenvironment and the degree of infiltration of immune cells are the key factors affecting the tumor immune response. It has been shown that tumors with pre-existing lymphocytes-infiltrating microenvironment are more likely to respond to immune-checkpoint inhibition (14). Immunotherapies are generally more effective in immunologically “hot” tumors, where abundant immune cells are already primed against tumor cells. However, “cold” tumors, which lack sufficient immune infiltration, are often less responsive to immunotherapy (15–17). Hence, tumors with high expression of *CXCR2P1* may have more active tumor immune microenvironment, which laid a foundation for good immune response.

Previous studies have shown that M1 macrophages and *CD4*+T cells can recruit more immune cells by secreting pro-inflammatory cytokines and chemokines, presenting antigens, and inducing the development and maturation of other immune cells in the tumor immune microenvironment, thereby playing an important role in tumor’s immune surveillance (18). Our research found that the expression of *CXCR2P1* was positively correlated with the number of M1 macrophages and activated *CD4*+ T cells in the tumor microenvironment. The reshaping of the immune microenvironment and the influence of *CXCR2P1* on the response to immunotherapy may be achieved through these two types of cells.

Tumor-associated macrophages (TAMs) are crucial in shaping the tumor immune microenvironment. Based on function and phenotype, macrophages can be divided into M1 and M2 types. M1 polarization is associated with tissue inflammation, mediating tissue damage and recruiting CD8+ T cells to target tumor cells. In contrast, M2 polarization is linked to anti-inflammatory responses, which facilitate tissue repair. The M1/M2 ratio in macrophages significantly influences the tumor immune microenvironment and tumor response to immunotherapy (19, 20). TAM molecular phenotypes are shaped by chemokines, cytokines, cell metabolites, and cross-talk signaling pathways in the tumor microenvironment (21). Activation of toll-like receptor (22), interferon (23), and *CD40* signaling pathways (24) promotes M1 macrophage differentiation, enhancing antigen presentation, directly phagocytosing tumor cells, and recruiting *CD8*+ T cells and NK cells (21). Conversely, TGF-β (25), γ-aminobutyric acid (26), and activation of myeloid checkpoint pathways (27, 28) shift macrophages toward an M2 phenotype, leading to IL-10 secretion and suppression of cytotoxic *CD8*+ T cells. Recent studies indicate that TAMs can also express PD-1, broadening the concept that PD-1/PD-L1 is exclusive to T cells’ immune escape mechanisms (29). This insight provides a potential explanation for *CXCR2P1*’s impact on PD-1 inhibitor responses via TAMs. Our study revealed a significant positive correlation between *CXCR2P1* expression and acute inflammatory responses, with notable activation of MHC II antigen presentation complexes and reduced gabaergic neurotransmitter secretion in tumors with high *CXCR2P1* expression. These findings suggest that *CXCR2P1* fosters M1 macrophage polarization within the tumor immune microenvironment, enhancing the tumor’s responsiveness to immunotherapy.

Through cytokine secretion and direct interactions with immune and tumor cells, *CD4*+ T cells play a pivotal role in the tumor immune response by influencing the composition and activity of the tumor immune microenvironment (30). In the “afferent” phase of the immune response, naive *CD4*+ T cells are stimulated by tumor-associated antigens presented by antigen-presenting cells. This stimulation triggers their differentiation into subsets such as T_H_1, T_H_2, T_H_17, T_H_9, follicular helper T cells (T_FH_), and regulatory T cells (T_reg_) (31, 32). Differentiated *CD4*+ T cells then migrate to tumor tissues or adjacent lymphoid tissues to participate in tumor immune responses (33). In the tumor immune microenvironment, *CD4*+ T cells can stimulate and maintain the differentiation of macrophages, *CD8*+ T cells and NK cells by secreting cytokines such as IFN-γ, TNF and IL-2, and inhibit angiogenesis of tumor, thus playing an anti-tumor effect (34–36). At the same time, *CD4*+ T cells can activate antigen-presenting cells through *CD40*-*CD40L* pathway to enhance the antigen presentation of tumor-associated antigens (37, 38). Recent studies have identified cytotoxic *CD4*+ T cells (activated T_H_1 cells) that directly kill tumor cells by releasing granzyme B and perforin (39). However, not all *CD4*+ T cells contribute to anti-tumor immunity. Subsets such as T_FH_ cells and T_reg_ cells can inhibit immune responses by expressing PD-L1 and secreting TGF-β, thus fostering tumor progression (40, 41).

The balance and composition of these *CD4*+ T cell subsets critically influence tumor immunogenicity. In our study, tumor tissues with high *CXCR2P1* expression exhibited significantly higher proportions of activated *CD4*+ T cells and T_FH_ cells compared to low *CXCR2P1* expression tumor, suggesting that the tumor tissues with high *CXCR2P1* expression may have higher immunogenicity. In addition, an increase in the proportion of follicular helper T cells indicates increased expression of PD-L1 in the tumor, which underlies the favorable response to PD-1 inhibitors (42). It has been shown that the degree of *CD4*+T cell infiltration in a tumor is directly related to a favorable patient outcome regardless of immunotherapy. However, how PD-1 inhibitor affects the phenotype of *CD4*+T cells in the immune microenvironment and how *CD4*+T cells enhance the response of tumor tissues to immune responses are still unclear, and further studies are needed to clarify the cellular biological mechanisms.

At the molecular level, *CXCR2P1* can activate immune-related signaling pathways and significantly increase the expression of PD-L1 in tumor tissues. Previous studies have demonstrated that increased levels of cytokines like IFN-γ (43) in the tumor microenvironment, along with the activation of the JAK-STAT (44), PI3K-Akt (45), and Toll-like receptor signaling pathways (46) can upregulate PD-L1 expression in both tumor and some immune cells, which enables tumor cells to evade immune surveillance (47). Activation of the PD-1 receptor further dampens the immune response by inhibiting key pathways in T cell receptor (TCR) signaling, such as the calcium signaling pathway (48) and MAPK signaling pathways (49), which leads to the reduction of IL-2 secretion and suppression of T cell activation.

In this study, we observed a significant positive correlation between the expression of *CXCR2P1* and PD-L1 in tumor tissues. GSEA and co-expression analysis revealed that tumor tissues with high *CXCR2P1* expression exhibited significant activation of the PD-1 signaling pathway, IFN signaling pathway, Toll-like receptor signaling pathway, and JAK-STAT signaling pathway. Conversely, the calcium signaling and MAPK pathways, integral to TCR signaling, were significantly suppressed. These findings suggest that *CXCR2P1* plays a dual role in modulating the tumor immune microenvironment. By enhancing antigen presentation, *CXCR2P1* may indirectly upregulates PD-L1 expression in tumor tissues by promoting immune cell infiltration. Elevated PD-L1 expression, coupled with enriched immune cell infiltration, enhances the tumor’s responsiveness to PD-1 inhibitors, contributing to favorable immunotherapy outcomes.

From GSEA analysis, we found *CXCR2P1* was enriched into microRNAs in tumor microenvironments, which indicated that *CXCR2P1* might affect the cell phenotype through microRNAs. Similar results was obtained by Zhao et al., who comprehensively analyzed the RNA transcriptome of head and neck squamous cell carcinoma and constructed a competing endogenous RNA network that significantly affected tumor immune infiltration through differential expressed genes analysis, finding that *CXCR2P1* may affect the OS of patients through the lncRNA-microRNA-mRNA axis (8). Our research firstly identified *MIR215* as the mediator of *CXCR2P1* affecting cell phenotype.

*MIR215* is an intragenic microRNA, whose host gene, *isoleucyl-tRNA synthetase 2 (IARS2)*, is a form of alanyl-tRNA synthetase (50). Previous researches had proved that *MIR215* can mediate the post-transcriptional gene silencing through receptor-interacting serine/threonine-protein kinase 1 (51), and hsa-miR-215-5p can be regulated by many lncRNAs, such as *CDC6* (52), *FTX* (53) and *UICLM* (54). *MIR215* is critically involved in regulating cell proliferation, apoptosis, and migration. Studies have shown that *MIR215* can promote the proliferation, migration, and invasion of gastric cancer cells (55). Its expression is significantly upregulated in gastric cancer tissues compared to normal gastric mucosa, with even higher levels observed in patients with poorly differentiated tumors, advanced clinical stages (stage III or IV), or lymph node metastases (56, 57). *Runt-related Transcription Factor 1 (RUNX1)* has been identified as a direct target of *MIR215* (58). In gastric cancer, *RUNX1* inhibits malignant cell proliferation by regulating the expression of key genes such as p21 and p53, thereby suppressing cell cycle progression and promoting cellular differentiation (59). The post-transcriptional silencing of *RUNX1* by *MIR215* might contribute to the enhanced invasive and proliferative capacities of tumor cells. Comprehensively, our findings suggest that high *CXCR2P1* expression may reduce hsa-miR-215-5p levels in tumor tissues through the lncRNA-microRNA regulatory axis, potentially improving the prognosis of gastric cancer patients. However, the precise role of *MIR215* in the immune microenvironment of tumor remains unclear, further studies are required to elucidate its underlying cellular and molecular mechanisms.

Our study has several limitations. First, as the only database focusing on the response of gastric cancer to PD-1 inhibitor, project PRJEB25780 is limited by its small sample scale, which may reduce the statistical robustness of our findings. Second, all conclusions drawn in this study are based on bioinformatic analyses, lacking validation through experimental approaches. Finally, due to the inherent limitations of bioinformatics analysis, the regulatory factors involved in the upstream and downstream signaling pathways of *CXCR2P1* remain unclear. Future studies should prioritize functional validation of *CXCR2P1* through *in vitro* or *in vivo* models, systematically map its upstream regulators and downstream signaling cascades, and assess its clinical relevance in larger immunotherapy cohorts with matched genomic and transcriptomic data.

## 5 Conclusion

In gastric cancer tissues, the expression of *CXCR2P1* is significantly elevated compared with normal gastric mucosa, and patients with high *CXCR2P1* expression exhibit better prognosis and improved responses to PD-1 inhibitor. *CXCR2P1* plays a vital role in reshaping the tumor immune microenvironment by promoting greater immune cell infiltration and significantly increasing the proportions of M1 macrophages, *CD4*+ T cells, and follicular helper T cells. At the cellular level, *CXCR2P1* is associated with inflammatory activation and enhanced antigen presentation in tumor tissues. At the molecular level, *CXCR2P1* upregulates PD-L1 expression in tumor tissues and activates key signaling pathways involved in PD-1 regulation. These effects might be mediated through the lncRNA-microRNA axis, with *MIR215* identified as a potential direct target of *CXCR2P1*. Future studies are required to experimentally validate the upstream and downstream signaling pathways of *CXCR2P1* and to further elucidate its cell biological mechanisms.

## Data Availability

The original contributions presented in the study are included in the article/supplementary material. Further inquiries can be directed to the corresponding authors.
